# A systematic review and meta-analysis of studies evaluating the performance and operational characteristics of dual point-of-care tests for HIV and syphilis

**DOI:** 10.1136/sextrans-2016-053069

**Published:** 2017-07-26

**Authors:** Harriet D Gliddon, Rosanna W Peeling, Mary L Kamb, Igor Toskin, Teodora E Wi, Melanie M Taylor

**Affiliations:** 1 London Centre for Nanotechnology, University College London, London, UK; 2 Department of Clinical Research, Faculty of Infectious and Tropical Diseases, London School of Hygiene and Tropical Medicine, London, UK; 3 Division of STD Prevention, Center for Disease Control and Prevention, Atlanta, Georgia, USA; 4 Department of Reproductive Health and Research, World Health Organization, Geneva, Switzerland

## Abstract

**Background:**

Mother-to-child transmission (MTCT) of syphilis and HIV continue to be important yet preventable causes of perinatal and infant morbidity and mortality.

**Objectives:**

To systematically review, critically appraise and perform a meta-analysis to evaluate the operational characteristics of dual rapid diagnostic tests (RDTs) for HIV/syphilis and evaluate whether they are cost effective, acceptable and easy to use.

**Design:**

Systematic review and meta-analysis.

**Data sources:**

We searched seven electronic bibliographic databases from 2012 to December 2016 with no language restrictions. Search keywords included HIV, syphilis and diagnosis.

**Review methods:**

We included studies that evaluated the operational characteristics of dual HIV/syphilis RDTs. Outcomes included diagnostic test accuracy, cost effectiveness, ease of use and interpretation and acceptability. All studies were assessed against quality criteria and assessed for risk of bias.

**Results:**

Of 1914 identified papers, 18 were included for the meta-analysis of diagnostic accuracy for HIV and syphilis. All diagnostic accuracy evaluation studies showed a very high sensitivity and specificity for HIV and a lower, yet adequate, sensitivity and specificity for syphilis, with some variation among types of test. Dual screening for HIV and syphilis was more cost effective than single rapid tests for HIV and syphilis and prevented more adverse pregnancy outcomes. Qualitative data suggested dual RDTs were highly acceptable to clients, who cited time to result, cost and the requirement of a single finger prick as important characteristics of dual RDTs.

**Conclusion:**

The results of this systematic review and meta-analysis can be used by policy-makers and national programme managers who are considering implementing dual RDTs for HIV and syphilis.

## INTRODUCTION

Approximately 1.5 million pregnant women annually are infected with HIV, and 900 000 are infected with syphilis.^1,2^ Mother-to-child transmission (MTCT) of HIV and syphilis remain significant causes of perinatal morbidity and mortality.^3^ HIV MTCT can occur during pregnancy, delivery or breastfeeding. Without any intervention, HIV MTCT rates vary between 20% and 35% in breastfed infants or 15% and 20% for non-breastfed infants.^4^ However, these MTCT rates for HIV can be reduced to less than 5% on provision of effective intervention.^5^ Untreated maternal syphilis results in in-utero infection, associated with significant adverse pregnancy outcomes, such as stillbirth, preterm and low birth weight, neonatal death and clinical syphilis infection in infants born alive.^6^ Systematic reviews indicate that in pregnant women with untreated syphilis, more than half of pregnancies result in these adverse outcomes,^7^ and that an even higher proportion of pregnancies are affected in women with primary or secondary syphilis infections.^8^ Prenatal syphilis screening followed by treatment with injectable penicillin early in pregnancy effectively treats the pregnant woman and prevents congenital syphilis. In addition, maternal syphilis has been shown to increase the risk of MTCT of HIV.^9^ The WHO launched a global initiative for elimination of congenital syphilis in 2007^8^ and has also prioritised the elimination of mother to child transmission (EMTCT) of HIV.^5^ Additionally in 2014, WHO HIV and STI programmes in collaboration with other UN partners joined forces to validate countries for the EMTCT of HIV and syphilis using shared guidelines and processes.^5,10^ Several countries have now achieved validation of EMTCT for HIV and/or syphilis.^11^

Screening all pregnant women for syphilis and HIV at first antenatal care visit is recommended in nearly all countries of the world and is being scaled up rapidly in countries committed to EMTCT of HIV and syphilis.^12,13^ However, while the testing of pregnant women for HIV is relatively well resourced, syphilis-infected pregnant women often go undiagnosed and untreated. While many countries have antenatal syphilis screening policies, more than 350 000 adverse pregnancy outcomes occur annually due to untreated maternal syphilis, despite the low cost of testing and treatment.^14^ To meet current targets, calls have been made to accelerate the dual EMTCT of syphilis and HIV.^15^ Early diagnosis and treatment of both HIV and syphilis in pregnant women has been proven as an effective strategy in the prevention of both adverse outcomes of pregnancy and MTCT. Key populations, such as men who have sex with men (MSM), transgender people, injecting drug users and sex workers would also benefit from improved HIV and syphilis screening coverage,^16–18^ as described in key policy documents published by the WHO.^19,20^

In 2015, the SD BIOLINE HIV/Syphilis Duo Test (Standard Diagnostics, Korea) was accepted for the WHO list of prequalified in vitro diagnostics.^21^ Other rapid diagnostics tests (RDTs) are also available that can simultaneously test for antibodies to HIV and *Treponema pallidum* antigens, ensuring that both tests can be conducted in a single visit to a single health facility. Herein, we describe a systematic review and meta-analysis of published literature to evaluate the operational characteristics of currently available RDTs for HIV and syphilis, including diagnostic accuracy, cost-effectiveness, acceptability and ease of test interpretation.

## METHODS

### Eligibility criteria

We followed the preferred reporting items for systematic reviews and meta-analysis (PRISMA) guidelines.^22^ Studies were included that evaluated, in either laboratory or field settings, any commercially available RDT (that satisfies the specifications in the ASSURED criteria^23,24^) that simultaneously tests for HIV and syphilis on the same cartridge or device. Studies were included that involved any sexually active populations in any geographic location. The primary outcome was diagnostic test accuracy (ie, sensitivity, specificity, positive predictive value, negative predictive value) for both HIV and syphilis. Secondary outcomes included cost-effectiveness, usability, ease of test interpretation and acceptability. The types of studies that were eligible for inclusion were evaluation studies, cost-effectiveness analyses and usability and acceptability studies. For the meta-analysis of diagnostic accuracy, studies were included if an acceptable reference standard for both HIV and syphilis was used (HIV: either enzyme immunoassay (EIA), Western blot (WB) or two RDTs; syphilis: *T. pallidum* particle agglutination assay (TPPA) or *T. pallidum* haemagglutination assay (TPHA) with or without non-treponemal testing). Studies were excluded if HIV and syphilis diagnosis were not conducted on a dual RDT (ie, on the same cartridge/device). Studies were included regardless of sample size.

### Search terms and strategy

We searched the following electronic bibliographic databases: Medline, Embase, KoreaMed, PAHO Library Catalogue, China National Knowledge Infrastructure, Russian Science Citation Index and J-stage. The search strategy included terms relating to HIV, syphilis and diagnosis (see online supplementary material). No language restrictions were used. Studies published between January 2012 and the December 2016 were sought. The searches were rerun immediately before the final analyses to check for recent relevant literature. Additional records were identified by searching bibliographies of relevant publications.

### Data extraction

Titles and abstracts were checked for relevance. For the meta-analysis of diagnostic accuracy, the data extracted included study title, dates of enrolment, country, test(s) evaluated, laboratory or field evaluation (and if so, sample type used), the population studied and for laboratory evaluations, whether fresh or archived specimens were used. For both the HIV and syphilis diagnosis components of each study, the following information was either extracted or calculated using two by two tables: the number of participants/samples used, prevalence (%), reference standard test, number of true positives, false positives, false negatives and true negatives. Study investigators were contacted if further information was required.

Two reviewers (HDG and MMT) independently extracted data from the included studies. Disagreements were resolved by consensus or by consulting external advisors. The updated standards for the reporting of diagnostic accuracy studies (STARD) checklist^25,26^ was used to evaluate the methodology of included studies. To critically appraise the included evaluation studies, the quality assessment of diagnostic accuracy studies (QUADAS-2) checklist^27^ was used.

### Data synthesis

Forest plots and summary receiver operating characteristic (SROC) curves were constructed using RevMan.^28^

## RESULTS

### Study characteristics

**Figure 1:**
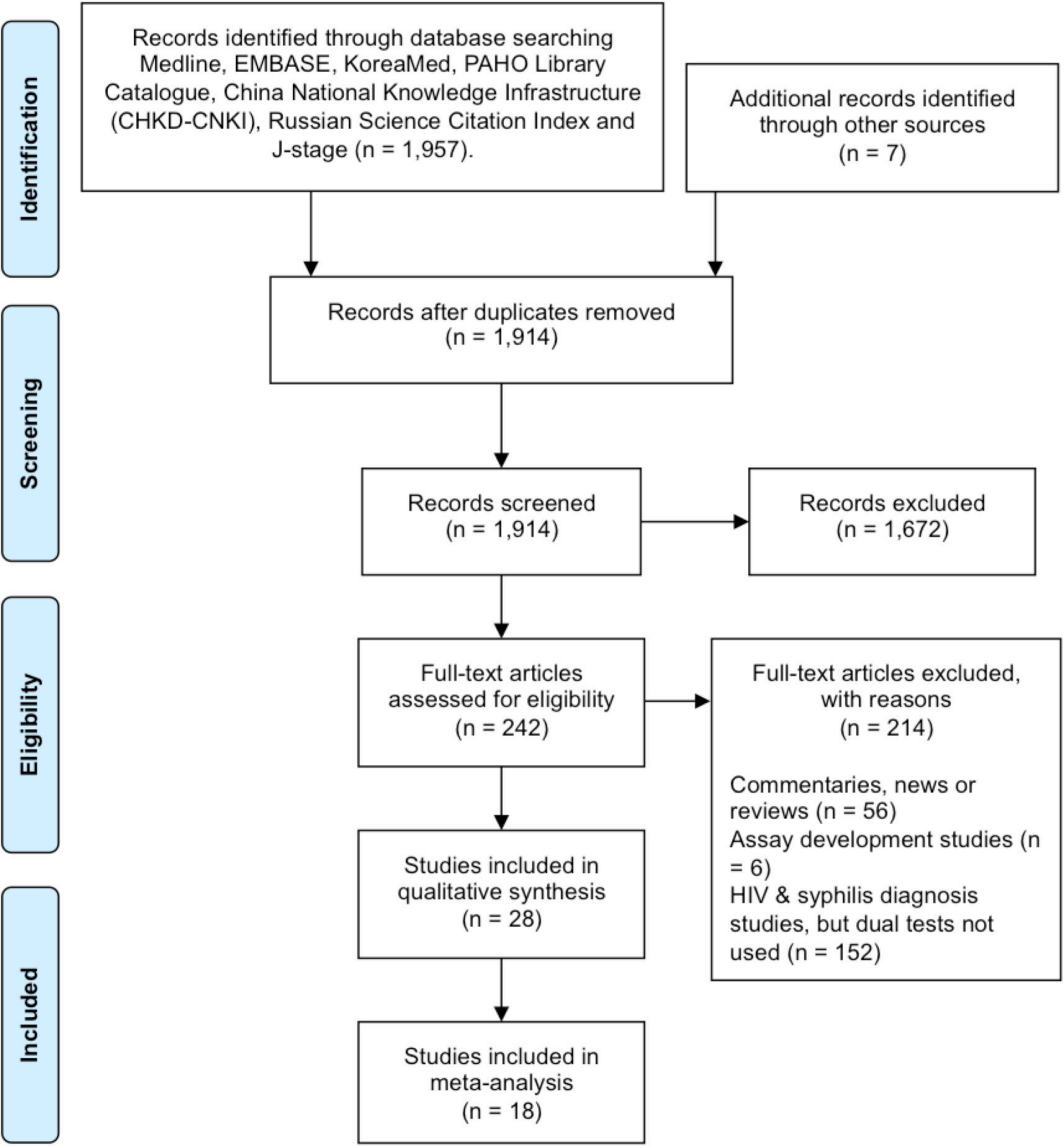
PRISMA flow diagram showing the number of records initially identified and that were subsequently excluded or included in the meta-analysis on the performance and operational characteristics of dual point-of-care tests for HIV and syphilis. PRISMA, preferred reporting items for systematic reviews and meta-analysis.

Among the 1914 records identified and screened (figure 1), we included 28 studies for the data synthesis, and 18 of these were also used in the meta-analysis. Two-by-two table data were not available for one study.^29^ Two studies included in the meta-analysis evaluated the performance of multiple tests. Diagnostic accuracy studies evaluated the performance of the SD BIOLINE HIV/Syphilis Duo Test, the MedMira Multiplo Rapid TP/HIV Antibody Test (MedMira, Canada) and the Chembio Dual Path Platform (DPP) HIV/Syphilis Assay (Chembio Diagnostic Systems, USA) (table s1). These studies were conducted in a range of WHO regions including Africa (South Africa, Kenya, Nigeria, Malawi, Ghana, Togo), South-East Asia (Nepal, Myanmar), the Western Pacific (China, Lao People’s Democratic Republic) and the Americas (Haiti, Peru, Mexico, USA). The populations studied were primarily key populations (such as sex workers, injection drug users (IDUs), transgender women, MSM and sexual health clinic attendees). Three studies evaluated the diagnostic accuracy of the test in antenatal care settings.

### Included and excluded studies

The 18 diagnostic accuracy studies that were included in the meta-analysis for diagnostic accuracy are detailed in table 1. The median sample sizes were 415 and 450 for HIV and syphilis, respectively, with the range for each falling between 150 and 10 000.^30–47^

One study was identified that evaluated the diagnostic accuracy of the INSTI Multiplex downward-flow immunoassay^48^ (also called the INSTI Multiplex HIV-1/HIV-2/Syphilis Antibody Test) (bioLytical Laboratories, Canada). Using 200 archived serum specimens from high-risk individuals in Peru, the results of this study suggested a high sensitivity (100%, 95% CI 95.9% to 100%) and specificity (95.5%, 95% CI 89.9% to 98.5%) for HIV diagnosis and a slightly lower sensitivity (87.4%, 95% CI 81.4% to 92.0%) but a higher specificity (97%, 95% CI 84.2% to 99.9%) for syphilis diagnosis. These results were not included in the meta-analysis because only one diagnostic accuracy evaluation study for this diagnostic test was identified. A study published by Leon *et al*^40^ evaluated visual interpretation of the Chembio DPP HIV/Syphilis Assay compared with the use of an electronic reader to interpret the test.^40^ The sensitivity and specificity for the HIV component of the Chembio DPP HIV/Syphilis Assay did not alter according to whether visual interpretation or electronic reader was used. The sensitivity of the syphilis component was similarly unaffected, but the specificity was slightly lower when the electronic reader was used (99.7%, 95% CI 98.2% to 100%) compared with visual interpretation (100%, 95% CI 98.8% to 100%), although this was not statistically significant. Results for test interpretation using the electronic reader were not included in the meta-analysis.

Hess *et al*^32^ studied the performance of the Chembio DPP HIV/Syphilis lateral flow assay in its original configuration (in which the liquid first flowed across the syphilis test line, followed by the HIV test line), and also in a revised or ‘reversed’ configuration (HIV followed by syphilis). This revised form of the test became the final approved model of the test. The Hess *et al*^32^ study reported two sets of sensitivities and specificities for the original order of the test and the reverse order. Only the results of the reverse order (which has since become the standard order for the test) were included in the meta-analysis. The study by Hess *et al*^32^ also assessed the performance of an integrated test for HIV, hepatitis C virus (HCV) and syphilis on a single diagnostic platform (Chembio DPP HIV-HCV-Syphilis Assay); but these results were not included in the meta-analysis because no other studies were identified that evaluated this particular RDT.

**Figure 2:**
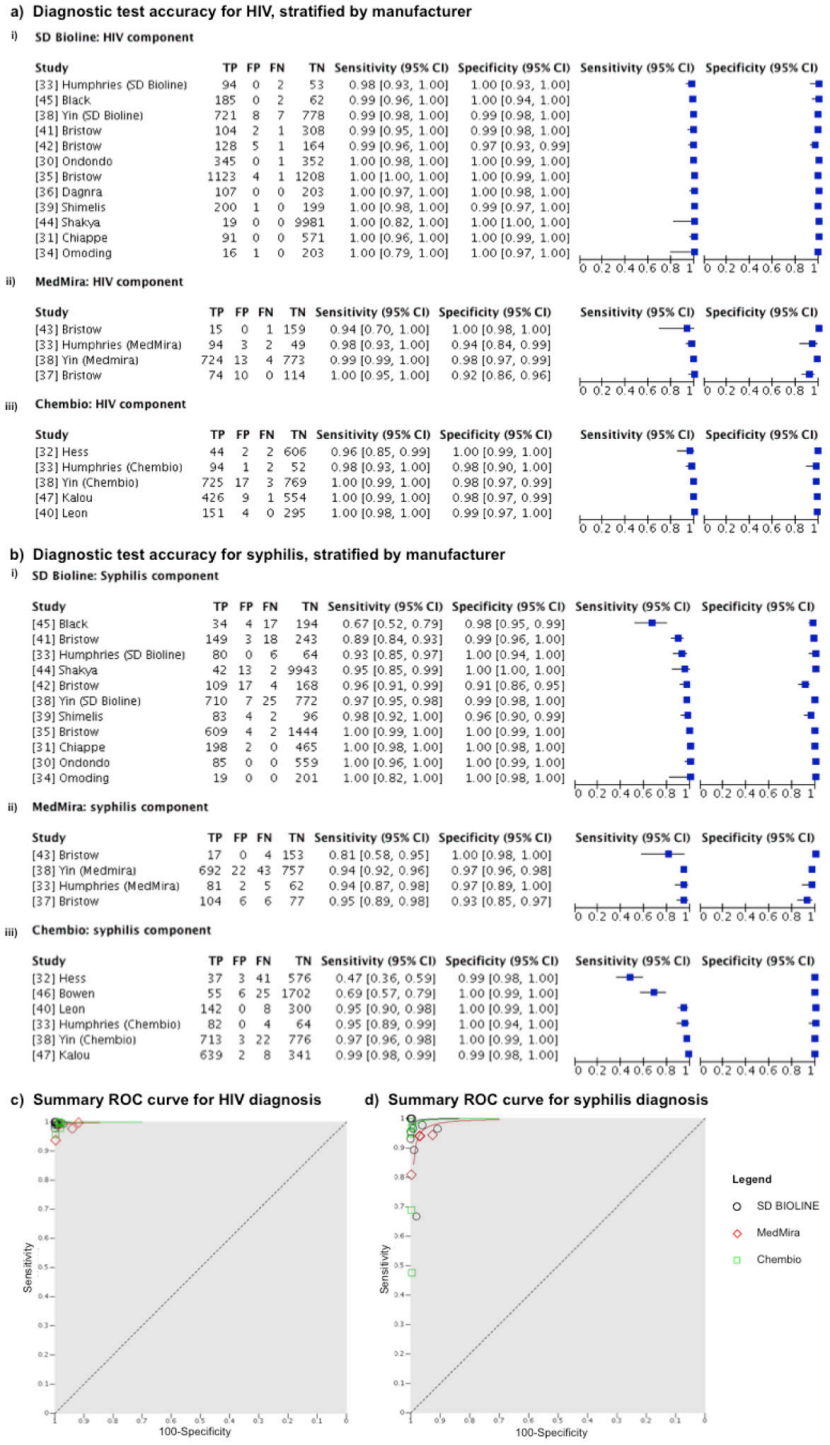
Meta-analysis of the diagnostic accuracy of dual HIV/syphilis RDTs, stratified according to manufacturer. Forest plots are sown for the diagnostic accuracy of (A) HIV and (B) syphilis diagnosis. Summary ROC curves are shown for the diagnosis of (C) HIV and (D) syphilis. RDTs, rapid diagnostic tests; ROC, receiver operating characteristic.

**Figure 3:**
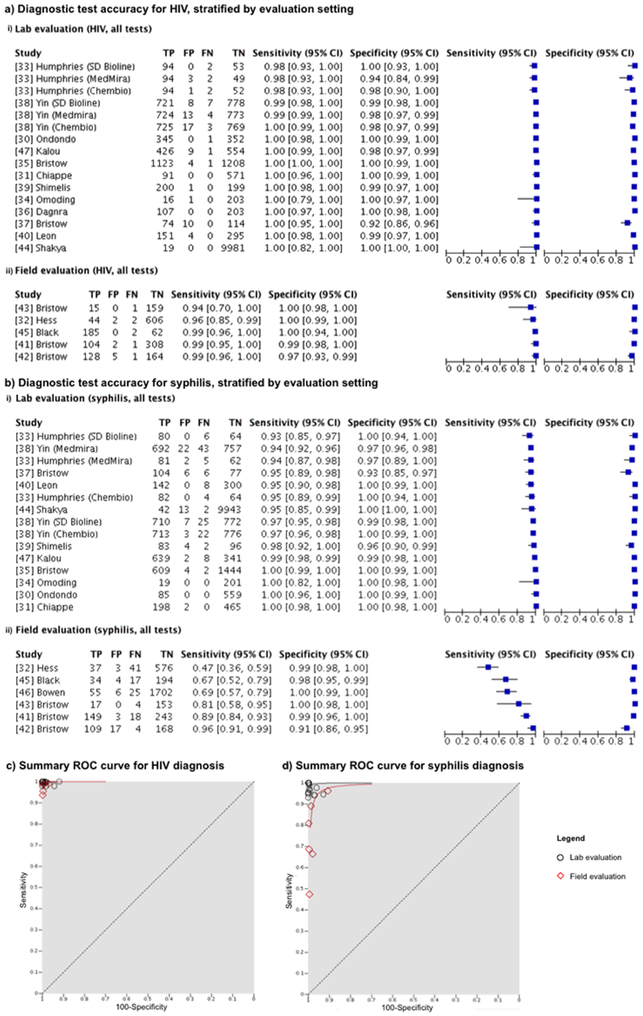
Meta-analysis of the diagnostic accuracy of dual HIV/syphilis RDTs, stratified according to the setting in which the evaluation was conducted. Forest plots are shown for the diagnostic accuracy of (A) HIV and (B) syphilis diagnosis. Summary ROC curves are shown for (C) HIV and (D) syphilis diagnosis. RDTs, rapid diagnostic tests; ROC, receiver operating characteristic.

**Figure 4:**
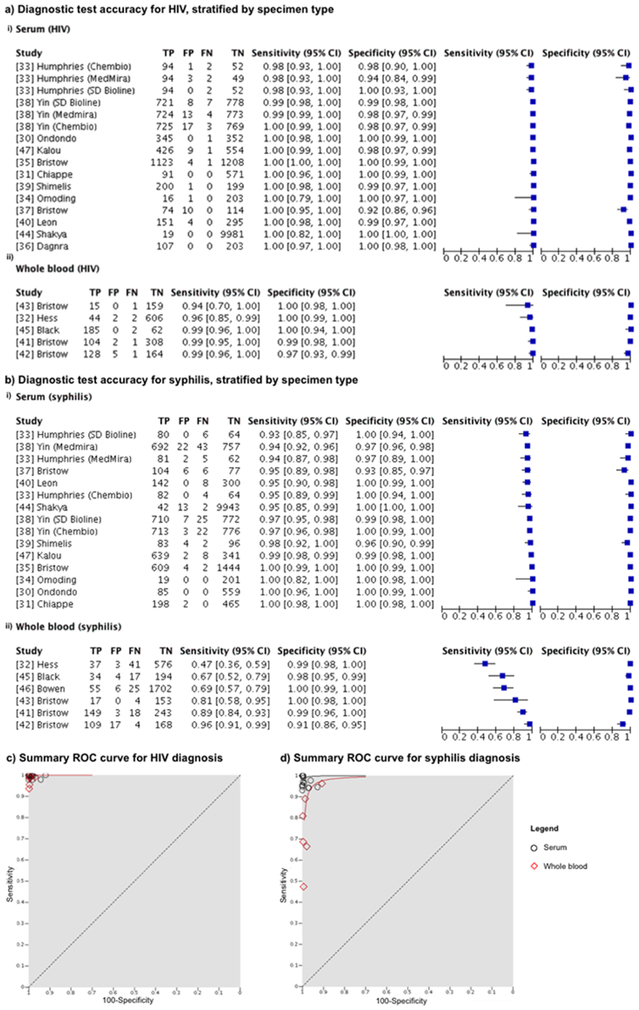
Meta-analysis of the diagnostic accuracy of dual HIV/syphilis RDTs, stratified according to the specimen type (serum or whole blood) used in the evaluation studies. Forest plots are shown for the diagnostic accuracy of (A) HIV and (B) syphilis diagnosis. Summary ROC curves are shown for (C) HIV and (D) syphilis diagnosis. RDTs, rapid diagnostic tests; ROC, receiver operating characteristic.

**Figure 5:**
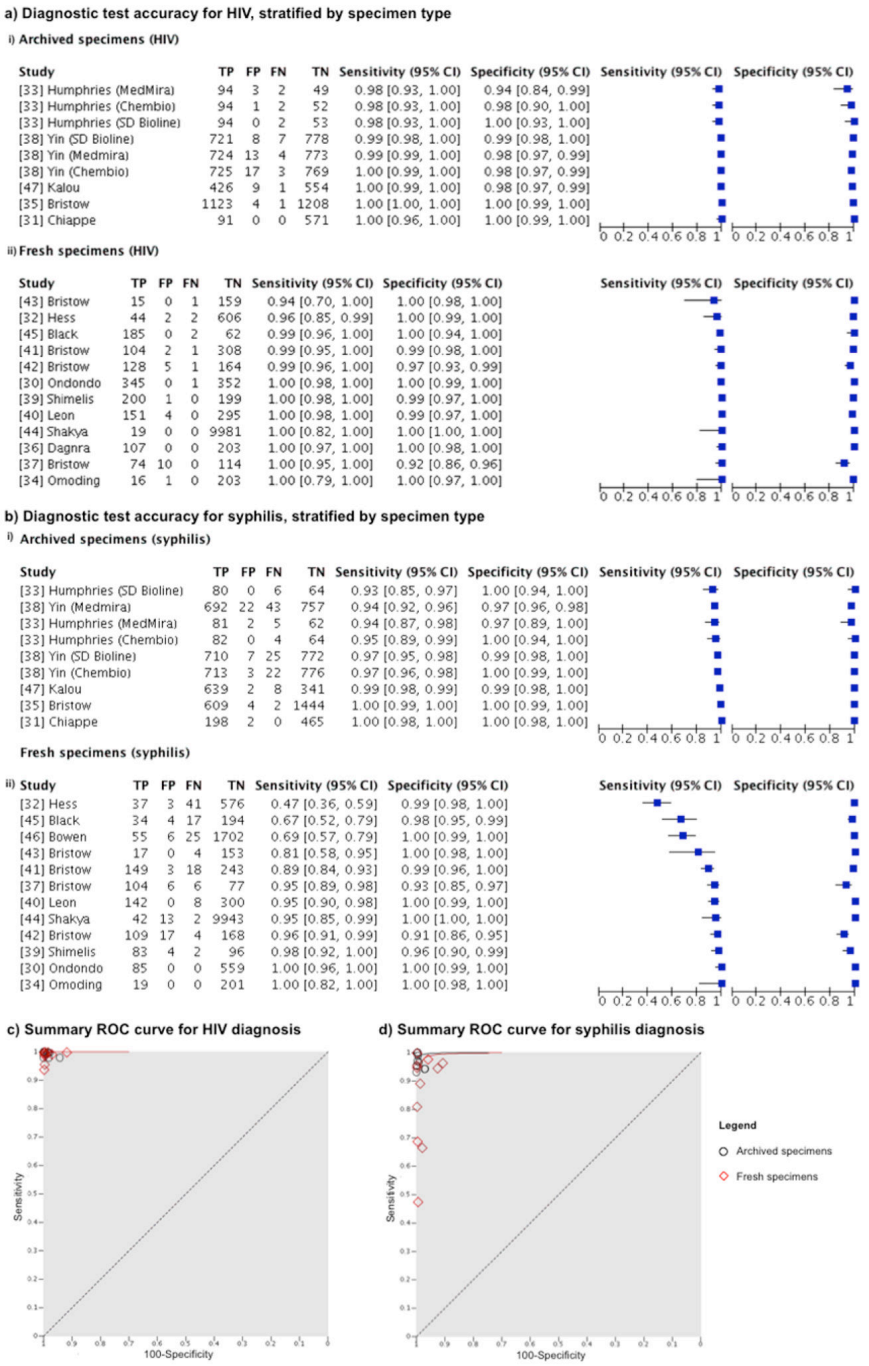
Meta-analysis of the diagnostic accuracy of dual HIV/syphilis RDTs, stratified according to the specimen type (archived, ie, frozen specimens or fresh specimens). Forest plots are shown for the diagnostic accuracy of (A) HIV and (B) syphilis diagnosis. Summary ROC curves are shown for (C) HIV and (D) syphilis diagnosis. FN, false negative; FP, false positive; RDTs, rapid diagnostic tests; ROC, receiver operating characteristic; TN, true negative; TP, true positive.

The meta-analysis stratification strategy is detailed in online supplementary figure 1. Tests were first stratified by manufacturer (figure 2), by evaluation setting (laboratory or field) (figure 3) and by specimen type used for evaluation (including serum versus whole blood, and archived versus fresh specimens) (figures 4 and 5).

### Diagnostic accuracy of HIV and syphilis by RDT manufacturer

#### HIV diagnostic performance by manufacturer

The diagnostic accuracy for HIV and syphilis of RDTs produced by three different manufacturers are detailed in figure 2. There were 12 studies that evaluated the SD BIOLINE HIV/Syphilis Duo Test, four studies that assessed the MedMira Multiplo Rapid TP/HIV Antibody Test and six that appraised the Chembio DPP HIV/Syphilis Assay, although one of these, reported by Bowen *et al*^46^ only reported the accuracy of syphilis diagnosis.

All but one of the evaluation studies reported a sensitivity of HIV diagnosis of at least 98%. This study, by Bristow *et al*^43^ reported a sensitivity of 94% for the MedMira Multiplo Rapid TP/HIV Antibody Test. The specificity values for HIV diagnosis ranged from 97% to 100% for the SD BIOLINE HIV/Syphilis Duo Test and 92% to 100% for the MedMira Multiplo Rapid TP/HIV Antibody Test. All of the specificity values for HIV diagnosis reported for the Chembio DPP HIV/Syphilis Assay were 100% (figure 2A).

The summary ROC curves for the three test manufacturers are presented in figure 2C, and D. Summary HIV ROC curve for the MedMira Multiplo Rapid TP/HIV Antibody test falls slightly below that of the curve for the SD BIOLINE HIV/Syphilis Duo Test and Chembio DPP HIV/Syphilis Assay, indicating that this test might have a lower diagnostic performance for HIV (figure 2C).

### Syphilis diagnostic performance by manufacturer

For syphilis diagnosis, the reported sensitivities for the SD BIOLINE HIV/Syphilis Duo Test were all between 89% and 100%, except for one study published by Black *et al*^45^ which reported a sensitivity of 67%. The authors of this study noted that patients with a rapid plasma regain (RPR) titre of ≥1:4 (an indicator of active syphilis) were more likely to test positive using this RDT. The specificity values reported for syphilis diagnosis using SD BIOLINE HIV/Syphilis Duo Test ranged from 91% to 100% (figure 2B).

The ranges for sensitivity and specificity reported for the syphilis component of MedMira Multiplo Rapid TP/HIV Antibody Test were 81% to 95% and 93% to 100%, respectively.

Chembio DPP HIV/Syphilis Assay gave sensitivity ranges for syphilis diagnosis of 46% to 97%, although each evaluation study reported a specificity of 100%. Similar to the study by Black *et al*^45^ of the SD Bioline HIV/Syphilis Duo Test syphilis component, Bowen *et al*^46^ also reported in their study of the Chembio DPP HIV/Syphilis Assay that patients with a high RPR titre (≥1:4) were more likely to test positive for presence of treponemal antibody.^46^

The summary ROC curve in figure 2D shows that SD BIOLINE HIV/syphilis Duo Test gives the highest syphilis diagnostic accuracy, followed by the Chembio DPP HIV/Syphilis Assay and then the MedMira Multiplo Rapid TP/HIV Antibody Test. However, these differences are not statistically significant.

### Diagnostic accuracy for HIV and syphilis in laboratory and field settings

Diagnostic accuracy results were also stratified according to whether the evaluation study was conducted in a laboratory or field setting (figure 3). Field evaluations were typically conducted in sexual health facilities,^32,41,42,45^ including one that actively recruited pregnant women.^42^ Another was conducted in antenatal settings^46^ and another at outreach sites for key populations.^43^ Results were combined, regardless of brand name or manufacturer.

#### HIV diagnostic performance in lab and field settings

In laboratory settings, the sensitivity of HIV diagnosis ranged from 94% to 99%, and specificity from 92% to 100%. In field settings, reported HIV sensitivity values were between 96% and 99% for all but one of the field evaluations. The sensitivity of HIV diagnosis in the remaining study, published by Bristow *et al*^43^ was 94%. This study evaluated the MedMira Multiplo Rapid TP/HIV Antibody Test. The range for specificity of HIV diagnosis reported in field settings was 97% to 100%.

#### Syphilis diagnostic performance in lab and field settings

For syphilis diagnosis, reported sensitivities ranged from 93% to 100% in laboratory settings, whereas for field settings, they ranged from 47% to 96%. The sensitivity value of 47% for syphilis diagnosis was reported by Hess *et al*^32^ for the Chembio DPP HIV/Syphilis Assay. In this study, only 11% of positive TPPA cases were RPR reactive, suggesting that the majority of cases represented previously treated rather than active syphilis infection. The next two lowest sensitivity values were reported by Black *et al*^45^ (67%) and Bowen *et al*^46^ (69%) for the SD BIOLINE HIV/Syphilis Duo Test and the Chembio DPP HIV/Syphilis Assay, respectively. Both Black *et al*^45^ and Bowen *et al*^46^ also reported on results of TPPA+/RPR+ test results as a standard, distinguishing between RPR titres >1:4 and <1:4 as indicators of active (transmissible) syphilis infection and old or treated infections (less transmissible), respectively.^6^ Both found the syphilis component of the DPP had high sensitivity and specificity in TPPA-reactive samples with higher RPR titres.

Using TPPA positivity as the standard, specificity values for syphilis diagnosis for the Chembio DPP HIV/Syphilis Assay ranged from 93% to 100% and 91% to 100% for laboratory and field settings, respectively.

### Diagnostic accuracy of HIV and syphilis by specimen type

It is possible that sample composition (ie, whole blood or serum) and storage can affect the diagnostic accuracy of RDTs. Long-term storage of frozen serum can affect the stability of proteins and other constituents of the sample.^49^ To investigate the diagnostic accuracy according to whether evaluation studies used serum or whole blood, and archived or fresh specimens, results were stratified according to specimen type (figure 4 and figure 5). Results were combined, regardless of brand name or manufacturer.

#### HIV diagnostic performance by specimen type

Reported sensitivities for HIV diagnosis were lower for studies that used whole blood (94% to 99%) compared with those that used serum (98% to 100%). However, studies using whole blood reported higher specificities (97% to 100%) than those using serum (92% to 100%) (figure 4A), leading to similar plotting of SROC curves for studies using serum and whole blood (figure 4C).

For studies using archived specimens, the sensitivity of HIV diagnosis ranged from 98% to 100%, and specificity from 94% to 100%. When fresh specimens were used, HIV diagnosis sensitivity values were between 94% and 100%, and specificity values were between 97% and 100% (figure 5A). Diagnostic accuracy for HIV was therefore minimally affected by specimen type.

#### Syphilis diagnostic performance by specimen type

Diagnostic accuracy for syphilis appears to be higher in studies that used serum rather than whole blood, with improved sensitivity ranges being reported (between 93% and 100% for studies that used serum, compared with 47% and 96% for studies that used whole blood) and specificities (93% to 100% for studies using serum compared with 91% to 100% for studies that used whole blood) (figure 4B).

Studies that used archived specimens reported syphilis sensitivities and specificities ranging from 93% to 100% and 97% to 100%, respectively. The diagnostic accuracy for syphilis was slightly poorer when fresh specimens were used, with sensitivities falling between 47% and 100% and specificities between 91% and 100% (figure 5B). This could reflect the fact that the evaluations using archived specimens were conducted in laboratory settings.

### Secondary outcomes

#### Cost-effectiveness and impact on adverse pregnancy outcomes

A study by Bristow *et al*^50^ showed that dual HIV/syphilis RDTs are an efficacious means of reducing the number of adverse pregnancy outcomes compared with other screening algorithms. In this study, four screening algorithms were compared, including an HIV RDT only, dual HIV and syphilis rapid RDTs, single RDTs for both HIV and syphilis and finally, HIV and syphilis laboratory tests. Costs of prevention and care were calculated, showing that a dual HIV/syphilis rapid testing strategy was both the least costly (US$226.92 per pregnancy) and resulted in the fewest adverse pregnancy outcomes (15 370 per 100 000 pregnancies for dual HIV/syphilis testing compared with 15 820 for HIV rapid testing only, 15 779 for HIV rapid testing and syphilis laboratory testing and 15 778 for single, separate RDTs for HIV and syphilis).

#### Feasibility and acceptability

A qualitative study conducted among patients seeking STI, HIV testing or antenatal care in Haiti evaluated the importance of various factors for HIV and syphilis dual testing to patients.^51^ The majority of study participants cited cost as the most important factor, but also selected single finger prick sampling and time to result as important attributes for dual testing. Interestingly, pregnant women reported that they prioritised time to result over all other factors. In antenatal care (ANC) settings in Colombia, dual HIV/syphilis RDTs were shown to have similar acceptability values to patients compared with separate rapid tests for HIV and syphilis.^52^

From the service provider perspective, in both China and Nigeria, dual HIV/syphilis RDTs were found to be fairly easy or very easy to use and to interpret the results, as was reported by Yin *et al*.^38^ This study scored the SD BIOLINE HIV/Syphilis Duo Test, the Chembio DPP HIV/Syphilis Assay, and the MedMira Multiplo Rapid TP/HIV Antibody Test on a range of operational characteristics. The SD BIOLINE HIV/Syphilis Duo Test scored the highest out of the three, with significant advantages over other tests in clarity of kit instruction, ease of use, ease of interpretation of results and training time required.

#### Cost-effectiveness, ease of test interpretation and acceptability of individual rapid tests for simultaneous HIV and syphilis diagnosis

Limited data were available on the cost effectiveness, usability, ease of test interpretation and acceptability of single device dual tests for dual HIV/syphilis diagnosis. However, the following studies were identified, which provide data for these factors when syphilis and HIV were diagnosed at the same time, using individual RDTs.

A systematic review that evaluated the impact of introducing rapid syphilis testing (RST) in antenatal care settings on HIV and syphilis uptake and coverage showed that RST may increase both syphilis and HIV screening rates in antenatal care settings.^53^ Two studies cited by the review that supported this claim were conducted by Strasser *et al*^54^ in Uganda and Zambia, and by Fleming *et al*^55^ in ANC settings in rural Kenya. More recently, in Kampala, Uganda, the introduction of syphilis testing within integrated HIV-antenatal care settings was shown to be effective, feasible and successfully capitalised on programmes that have already been established and optimised for HIV care.^56^

The acceptability of simultaneous testing for HIV and syphilis using separate RDTs was investigated in key populations in Peru.^57^ The tests used were the SD BIOLINE HIV 1/2 3.0 and SD BIOLINE Syphilis 3.0 tests. Client perceptions and the feasibility of implementing simultaneous HIV and syphilis RDTs were evaluated. The proportion of clients tested who received timely results increased by 30.8% for HIV and 35.7% for syphilis in pregnant women. The RDTs for HIV and syphilis allowed for fewer hospital visits, less time spent waiting at the hospital and lower labour and resource costs for the hospital. All clients tested were either completely satisfied (52%) or satisfied (48%) with the process of simultaneous HIV/syphilis testing. Seventy-two per cent of study participants strongly agreed with the statement, ‘I liked the process of having the two tests taken at the same time’.

A study published by Owusu-Edusei *et al*^58^ simulated the cost-effectiveness of using separate laboratory-based diagnosis for HIV and syphilis in China. Their results suggested that incorporating syphilis screening into pre-existing antenatal HIV screening programmes was more cost-effective than HIV screening only even in very low prevalence settings, and that testing for both infections would prevent a larger number of adverse pregnancy outcomes.

### Quality of studies

The STARD checklist was used to appraise the quality of reporting of studies included in the meta-analysis (see online supplementary file 1). Of the 30 items in the updated STARD checklist published by Bossuyt *et al*^26^ some items were universally well reported, such as the identification as a study of diagnostic accuracy (100%), scientific and clinical background (100%), index test and reference standard methods (100%), methods for estimating or comparing measures of diagnostic accuracy (94%) and implications for clinical practice (100%). However, other items were poorly reported, such as the rationale for choosing the reference standard where alternatives exist and whether clinical information and reference standard results were available to the performers of the index tests or if clinical information and index test results were available to assessors of the reference standard. In addition, few studies included a flow diagram of participants.

Quality of methodology was assessed using the QUADAS-2,^27^ and results are summarised in online supplementary table 3. Most studies either scored as low or unclear risk of bias where patient selection, index tests and study flow and timing were concerned. In particular, studies were reported as having an unclear risk of bias for the reference standard if they did not state that results of the reference standard were interpreted without knowledge of the results of the index tests. Little concern was identified regarding the applicability of patients, index tests and reference standards used in the studies.

## DISCUSSION

The overall purpose of this study was to evaluate the literature relating to dual RDTs for HIV and syphilis, particularly with regards to their diagnostic accuracy, cost-effectiveness, feasibility, acceptability and ease of interpretation. This meta-analysis reviewed the results of 18 recently published studies on the performance characteristics of rapid dual HIV/syphilis tests when evaluated by manufacturer and performance in field versus laboratory settings. The diagnostic accuracy for HIV was found to vary minimally depending on test manufacturer, with SD BIOLINE HIV/Syphilis Duo Test and the Chembio DPP HIV/Syphilis Assay showing the highest diagnostic accuracy. Diagnostic accuracy for syphilis varied with manufacturer, with the SD BIOLINE HIV/Syphilis Duo Test being the most accurate. Performance of the test in the laboratory versus field setting did not result in a difference in the sensitivity or specificity for HIV but a poorer sensitivity was noted in two field-based studies for syphilis. Published literature on the cost-effectiveness and feasibility of dual RDTs for HIV and syphilis was limited but demonstrated encouraging results that, along with performance results, could be used to support the use of these tests for screening of populations at risk for HIV and syphilis.

The diagnostic accuracy for HIV of each of the three dual tests (SD BIOLINE HIV/Syphilis Duo Test, MedMira Multiplo Rapid TP/HIV Antibody Test and the ChemBio DPP HIV/Syphilis Assay) evaluated in the meta-analysis was consistently high (with all studies but one reporting sensitivities of over 98% and all but two reporting specificities of over 97%). The diagnostic accuracy for HIV was found to be slightly lower for the MedMira Multiplo Rapid TP/HIV Antibody Test, which gave sensitivities of between 94% and 100% and specificities between 98% and 100%. In comparison, the SD BIOLINE HIV/Syphilis Duo Test and Chembio DPP HIV/Syphilis Assay gave higher levels of sensitivities, with a range of 98% to 100% for each. Their reported specificities were also higher, with 97%–100% for the SD BIOLINE HIV/Syphilis Duo Test and 98%–100% for the Chembio DPP HIV/Syphilis Assay. This was also true for syphilis diagnostic accuracy, with the SD BIOLINE HIV/Syphilis Duo Test performing the best out of the three. It should be noted that single RDTs for syphilis have also shown sensitivities between 64% and 100% and reduced sensitivities for clinic-based evaluations compared with laboratory evaluations.^54^ A target product profile (TPP) for an ideal dual HIV/syphilis RDT was developed at the 1st Technical Consultation on Point-Of-Care Diagnostic Tests for Sexually Transmitted Infections convened by the WHO Reproductive Health Research Department in May 2004.^59^ This TPP set out the desired operational characteristics for a dual HIV/syphilis RDT and included minimal and optimal specifications for parameters such as sensitivity and specificity of HIV and syphilis diagnosis. The minimal sensitivity and specificity specifications were >98% and >98% for HIV, respectively, and >85% and >95%, respectively, for syphilis. The three RDTs that were included in the meta-analysis all fulfil each of these requirements, at least at the minimal level. Minimum standards for HIV RDTs have been suggested to be >99% for sensitivity and >98% specificity.^60^

Accuracy of HIV diagnosis was minimally affected by conducting the test in the laboratory compared with field settings. However, a reduction in diagnostic accuracy was seen in field settings for syphilis. This was particularly true for the Chembio DPP HIV/Syphilis Assay. Two studies identified improvements in sensitivity when RPR titre values were included to identify active syphilis.^41–43,45,46^ This suggests that despite lower overall reported sensitivities, these tests may preferentially detect active syphilis over old or treated syphilis, which is clinically advantageous. The SD Bioline HIV/Syphilis Test has received WHO prequalification^21^ and is ready for use according to country-established quality performance measures. Our results indicate that, although the syphilis performance component as assessed by these studies still meets the minimal criteria as specified for the TPP, efforts to ensure consistent, correct and repeated staff training and quality control measures should be undertaken at the field level to ensure appropriate use and interpretation of these tests.

The overall diagnostic accuracy for HIV was minimally affected according to whether serum or whole blood was used in the evaluation studies. However, studies that used serum reported a superior diagnostic performance for syphilis than those that used whole blood. Specimen type (archived versus fresh specimens) was shown to marginally affect diagnostic accuracy for syphilis but not for HIV. These findings mimic those of the lab versus field analysis as all of the archived specimens would have been tested in a laboratory setting. However, the findings of the archived versus fresh analysis demonstrate the good performance of the RDTs on archived specimens.

Evidence from qualitative studies gives a strong indication that dual testing for HIV and syphilis is acceptable to testing clients, feasible for implementation in a range of ANC and other programmes and cost-effective. In a modelling study, when compared with HIV testing alone using a RDT, two separate RDTs for HIV and syphilis, and separate laboratory testing for HIV and syphilis, dual RDTs for HIV and syphilis were shown to be the least costly and also prevented the largest number of adverse pregnancy outcomes.^50^ A study conducted in Haiti assessed the importance of a range of RDT characteristics for testing clients. The most important factors included cost, the requirement for a single finger prick sample and time to result.^51^ Published literature on simultaneous testing for HIV and syphilis using separate RDTs also provided evidence on the feasibility of implementation of incorporating syphilis testing into pre-existing HIV screening programmes, acceptability and cost-effectiveness.^53,56–58^ Unfortunately, less data were available in the literature concerning ease of dual RDT interpretation.

Our meta-analysis has some limitations. First, our search criteria may have missed some studies and the authors are aware of ongoing studies for which results are not yet available. Second, to date only a limited number of diagnostic accuracy evaluation studies have been published for each dual RDT. While the best diagnostic performance was observed for the SD BIOLINE HIV/Syphilis Duo Test, more evaluation studies were available for this test compared with the two others included in the meta-analysis. More evidence is required to inform our understanding of the performance of the MedMira Multiplo Rapid TP/HIV Antibody Test and Chembio DPP HIV/Syphilis Assay. In particular, more field evaluation studies are warranted, in order to assess the sensitivity of the syphilis component of the diagnostic tests under the conditions and using operators that are likely available in real world versus controlled, laboratory settings. Third, studies used a range of reference tests with which to evaluate the diagnostic accuracy of the dual RDTs. For example, some studies used treponemal tests (measuring ever exposure to *T. pallidum*, regardless of previous treatment) only, whereas others used both treponemal and non-treponemal tests (measuring active infection). Where archived specimens were used, studies did not mention when reference testing was carried out (ie, at the time of collection or at the same time as the index test), and this timing could affect the agreement between the results of reference and index test. Another limitation is that for the studies that used archived specimens, it was unclear what population these specimens were taken from and in what setting. Too few studies were available for a reliable performance comparison of the RDTs with use of treponemal and non-treponemal tests (n=4) versus treponemal only as reference standards for syphilis diagnosis. This analysis may be considered for future study. Finally, only three commercially available dual HIV/syphilis, RDTs were evaluated in this meta-analysis. However, other tests are available which we did not include in this analysis, such as the INSTI Multiplex HIV-1/HIV-2/Syphilis Antibody Test, and there are more in development, such as the mChip, a smartphone dongle that performs an ELISA on a small chip using microfluidics.^61^

Our results demonstrate excellent performance for the HIV component of the dual RDTs for HIV and syphilis and good performance for the syphilis component, similar to the performance of single syphilis RDTs.^54,62^ When considering performance, cost-effectiveness and feasibility, these tests should be prioritised for use in settings and among populations where HIV and syphilis screening are recommended, namely, antenatal care settings. As the screening recommendations for HIV and syphilis are similar in many respects, it is logical and practical to combine their diagnosis on a single cartridge.^63^ Dual RDTs for HIV and syphilis testing would allow same-day treatment for syphilis and immediate referral for HIV therapy, thus enhance the prevention of MTCT of HIV and syphilis.^64^ Dual RDTs for HIV and syphilis therefore represent an important measure in the elimination of MTCT of HIV and syphilis. This systematic review will inform the WHO process for developing diagnostic algorithms for the use of dual RDTs for HIV and syphilis diagnosis. In the interim, WHO has developed interim guidance for use and interpretation of these tests.^65^ Future work will be required to build a toolkit for programme and clinic managers, similar to the one that was established for rapid syphilis testing,^66^ which would provide guidance on planning, management and implementation of dual RDTs for HIV and syphilis.

## Supplementary Material

Supplemental

## Figures and Tables

**Table 1:** Characteristics and results of studies evaluating the diagnostic test accuracy of dual HIV/syphilis RDTs that were included in the meta-analysis according to order of date published

						HIV								Syphilis							
Author and year	Location	Population	Study setting (lab or field)	Index test	Sample	Reference test	TP	FP	FN	TN	Sens (%)	Spec (%)	Prev (%)	Reference test	TP	FP	FN	TN	Sens (%)	Spec (%)	Prev (%)
Ondondo^30^ 2013	Kenya	HIV serodiscordant couples	Lab	SD BIOLINE HIV/Syphilis Duo Test	Serum	2 RDTs, confirmed by 2 EIAs	345	0	1	352	99.7	100.0	49.6	RPR, confirmed by TPHA	85	0	0	559	100.0	100.0	12.2
Chiappe^31^ 2013	Peru	Archived specimens	Lab	SD BIOLINE HIV/Syphilis Duo Test	Serum	EIA and WB	91	0	0	571	100.0	100.0	13.7	RPR, confirmed by TPPA	198	2	0	465	100.0	99.6	29.8
Hess^32^ 2014	California, USA	STD clinic attendees, IDUs, MSM	Field	Chembio DPP HIV/Syphilis Assay (reverse order)	Whole blood	EIA	44	2	2	606	95.7	99.7	6.7	TPPA	37	3	41	576	47.4	99.5	10.6
Humphries^33^ 2014	California, USA	Archived specimens	Lab	SD BIOLINE HIV/Syphilis Duo Test	Serum	EIA and WB	94	0	2	53	97.9	100.0	64.0	TPPA	80	0	6	64	93.0	100.0	57.3
Humphries^33^ 2014	California, USA	Archived specimens	Lab	Chembio DPP HIV/Syphilis Assay	Serum	EIA and WB	94	1	2	52	97.9	98.1	64.0	TPPA	82	0	4	64	95.3	100.0	57.3
Humphries^33^ 2014	California, USA	Archived specimens	Lab	MedMira Multiplo Rapid TP/HIV Antibody Test	Serum	ElA and WB	94	3	2	49	97.9	94.2	64.0	TPPA	81	2	5	62	94.1	96.9	57.3
Omoding^34^ 2014	Uganda	Pregnant women	Lab	SD BIOLINE HIV/Syphilis Duo Test	Serum	3 RDTs	16	1	0	203	100.0	99.5	7.3	TPHA	19	0	0	201	100.0	100.0	8.6
Bristow^35^ 2014	Ghana, Mexico, Lao People’s Democratic Republic, Togo, Kenya and Myanmar	Archived specimens from STI clinic attendees	Lab	SD BIOLINE HIV/Syphilis Duo Test	Serum	EIA, WB and RDTs	1123	4	1	1208	99.9	99.7	48.1	TPPA/TPHA/EIA	609	4	2	1444	99.7	99.7	29.7
Dagnra^36^ 2014	Togo	Key populations	Lab	SD BIOLINE HIV/Syphilis Duo Test	Serum	EIA	107	0	0	203	100.0	100.0	34.5	/	/	/	/	/	/	/	/
Bristow^37^ 2015	Peru	MSM and transgender women	Lab	MedMira Multiplo Rapid TP/HIV Antibody Test	Serum	EIA and WB	74	10	0	114	100.0	91.9	37.4	TPPA	104	6	6	77	94.6	92.8	57.0
Yin^38^ 2015	Nanjing, China, Zaria, Nigeria and Ibadan, Nigeria	Archived samples	Lab	SD BIOLINE HIV/Syphilis Duo Test	Serum	EIA	721	8	7	778	99.0	99.0	48.1	TPPA or TPHA	710	7	25	772	96.6	99.1	48.5
Yin^38^ 2015	Nanjing, China, Zaria, Nigeria and Ibadan, Nigeria	Archived samples	Lab	MedMira Multiplo Rapid TP/HIV Antibody Test	Serum	EIA	724	13	4	773	99.5	98.3	48.1	TPPA or TPHA	692	22	43	757	94.2	97.2	48.5
Yin^38^ 2015	Nanjing, China, Zaria, Nigeria and Ibadan, Nigeria	Archived samples	Lab	Chembio DPP HIV/Syphilis Assay	Serum	EIA	725	17	3	769	99.6	97.9	48.1	TPPA or TPHA	713	3	22	776	97.0	99.6	48.5
Shimelis^39^ 2015	Ethiopia	STI clinic attendees	Lab	SD BIOLINE HIV/Syphilis Duo Test	Serum	RDTs and EIA	200	1	0	199	100	99.5	50.0	TPHA	83	4	2	96	97.6	96.0	45.9
Leon^40^ 2016	Peru	MSM and transgender women	Lab	Chembio DPP HIV/Syphilis Assay	Serum	EIA and WB	151	4	0	295	100.0	98.7	33.6	TPPA	142	0	8	300	94.7	100.0	33.3
Bristow^41^ 2016	Peru	MSM and transgender women	Field	SD BIOLINE HIV/Syphilis Duo Test	Whole blood	EIA and WB	104	2	1	308	99.1	99.4	25.3	TPPA	149	3	18	243	89.2	98.8	40.4
Bristow^42^ 2016	Haiti	STI clinic attendees	Field	SD BIOLINE HIV/Syphilis Duo Test	Whole blood	RDTs	128	5	1	164	99.2	97.0	43.3	TPHA and ELISA	109	17	4	168	96.5	90.8	37.9
Bristow^43^ 2016	Peru	Sex workers, MSM and transgender women	Field	MedMira Multiplo Rapid TP/HIV Antibody Test	Whole blood	EIA and WB	15	0	1	159	93.8	100.0	7.8	TPPA	17	0	4	153	81.0	100.0	10.2
Shakya^44^ 2016	Nepal	Pregnant women	Lab	SD BIOLINE HIV/Syphilis Duo Test	Serum	3 RDTs	19	0	0	9981	100.0	100.0	0.2	RPR, confirmed by TPHA	42	13	2	9943	95.5	99.9	0.4
Black^45^ 2016	South Africa	Female sex workers	Field	SD BIOLINE HIV/Syphilis Duo Test	Whole blood	EIA	185	0	2	62	98.8	100.0	75.1	TPPA and RPR (titre ≥1:8)	34	4	17	194	66.7	98.0	20.5
Bowen^46^ 2016	Malawi	Pregnant women	Field	Chembio DPP HIV/Syphilis Assay	Whole blood	RDT	/	/	/	/	/	/	/	TPPA	55	6	25	1702	68.8	99.6	4.5
Kalou^47^ 2016	Georgia, USA	Archived specimens	Lab	Chembio DPP HIV/Syphilis Assay	Serum	EIA and WB	426	9	1	554	98.8	100.0	43.1	TPPA & EIA (Trep Sure)	639	2	8	341	98.8	99.4	65.4

EIA, enzyme immunoassay; FP, false positive; FN = false negative; IDU, injection drug user; MSM, men who have sex with men; Prev, prevalence; RDT, rapid diagnostic test; RPR, rapid plasma regain; Sens, sensitivity; Spec, specificity; TN, true negative; TP, true positive; TPHA*, Treponema pallidum* haemagglutination assay; TPPA, *T. pallidum* particle agglutination assay; WB, Western blot.

## Key messages

- In studies of dual HIV/syphilis tests on a single device, the accuracy of HIV diagnosis remained high regardless of test manufacturer or whether evaluations were conducted in laboratory or field settings.
- The accuracy of syphilis testing was good (similar to single tests for syphilis), although not as high as HIV, in both laboratory and field settings and regardless of manufacturer.
- Dual testing for HIV and syphilis has been shown to be more cost-effective and more effective at preventing adverse pregnancy outcomes than testing for HIV alone or using separate RDTs for HIV and syphilis.
