# Supplementary material for: A systematic review and meta-analysis of studies evaluating the performance and operational characteristics of dual point-of-care tests for HIV and syphilis
Source: Sex Transm Infect. Author manuscript; Available in PMC 2019 Sep 21. (PMC6754342; doi:10.1136/sextrans-2016-053069)
Supplement: Supplemental [file NIHMS1050480-supplement-Supplemental.docx]

**Search terms used for Medline Database**

(2012/01/01[PDAT] : 2016/10/03[PDAT]) AND

("Syphilis"[Mesh] OR Chancre [TW]OR Neurosyphilis [TW] OR " Tabes Dorsalis " [TW] OR syphilis [TW] OR "Treponema pallidum"[Mesh] OR "treponema pallidum" [TW]) AND

(“HIV Infections” [MeSH] OR “HIV”[MeSH] OR “hiv”[tw] OR “hiv-1”[tw] OR “hiv-2”[tw] OR “hiv1”[tw] OR “hiv2”[tw] OR hiv infect*[tw] OR “human immunodeficiency virus”[tw] OR “human immunedeficiency virus”[tw] OR “human immuno-deficiency virus”[tw] OR “human immune-deficiency virus”[tw] OR ((human immun*) AND (“deficiency virus”[tw])) OR “acquired immunodeficiency syndrome”[tw] OR “acquired immunedeficiency syndrome”[tw] OR “acquired immuno-deficiency syndrome”[tw] OR “acquired immune-deficiency syndrome”[tw] OR ((acquired immun*) AND (“deficiency syndrome”[tw])) OR "Sexually Transmitted Diseases, Viral"[MeSH:NoExp])

AND

(sensitiv*[Title/Abstract] OR sensitivity and specificity[MeSH Terms] OR diagnose[Title/Abstract] OR diagnosed[Title/Abstract] OR diagnoses[Title/Abstract] OR diagnosing[Title/Abstract] OR diagnosis[Title/Abstract] OR diagnostic[Title/Abstract] OR diagnosis[MeSH:noexp] OR diagnostic * [MeSH:noexp] OR diagnosis,differential[MeSH:noexp] OR diagnosis[Subheading:noexp] OR “point of care” [TW])

Evaluation studies of all dual HIV/syphilis RDTs

SD BIOLINE HIV/Syphilis Duo Test

MedMira Multiplo Rapid TP/HIV Antibody Test

Chembio DPP HIV/syphilis Assay

Evaluation studies of all dual HIV/syphilis RDTs

Laboratory evaluation

Field evaluation

a)

b)

Figure S1. Stratification strategy. (a) Evaluation studies were first stratified according to the RDT manufacturer and (b) according to whether the evaluation study was conducted in laboratory or field settings.

Evaluation studies of all dual HIV/syphilis RDTs

Archived specimens

All other specimens

c)

Table S1. Results of STARD evaluation for diagnostic test accuracy evaluation studies included in the meta-analysis.

| **Study ID** | **Author** | **Year** | **1** | **2** | **3** | **4** | **5** | **6** | **7** | **8** | **9** | **10** | **11** | **12** | **13** | **14** | **15** | **16** | **17** | **18** | **19** | **20** | **21** | **22** | **23** | **24** | **25** | **26** | **27** | **28** | **29** | **30** |
| --- | --- | --- | --- | --- | --- | --- | --- | --- | --- | --- | --- | --- | --- | --- | --- | --- | --- | --- | --- | --- | --- | --- | --- | --- | --- | --- | --- | --- | --- | --- | --- | --- |
| 30 | Ondondo^30^ | 2013 | 1 | 1 | 1 | 1 | 1 | 0 | 0 | 0 | 0 | 1 | 0 | 0 | 0 | 1 | 0 | 0 | 0 | 0 | 0 | 0 | 0 | 0 | 0 | 0 | 0 | 0 | 1 | 0 | 0 | 0 |
| 31 | Chiappe^31^ | 2013 | 1 | 1 | 1 | 1 | 0 | 0 | 0 | 0 | 0 | 1 | 0 | 0 | 1 | 1 | 0 | 0 | 0 | 0 | 0 | 0 | 0 | 0 | 0 | 0 | 0 | 0 | 1 | 0 | 0 | 0 |
| 32 | Hess^32^ | 2014 | 1 | 1 | 1 | 1 | 1 | 1 | 1 | 1 | 1 | 1 | 0 | 0 | 1 | 1 | 0 | 1 | 0 | 0 | 0 | 1 | 0 | 0 | 1 | 1 | 0 | 1 | 1 | 0 | 0 | 1 |
| 33 | Humphries^33^ | 2014 | 1 | 0 | 1 | 1 | 1 | 0 | 0 | 0 | 0 | 1 | 0 | 0 | 1 | 1 | 1 | 0 | 0 | 0 | 0 | 0 | 0 | 1 | 1 | 1 | 0 | 1 | 1 | 0 | 0 | 1 |
| 34 | Omoding^34^ | 2014 | 1 | 1 | 1 | 1 | 1 | 1 | 1 | 1 | 1 | 1 | 0 | 0 | 0 | 1 | 0 | 0 | 0 | 0 | 0 | 1 | 0 | 0 | 1 | 1 | 0 | 1 | 1 | 0 | 0 | 0 |
| 35 | Bristow^35^ | 2014 | 1 | 1 | 1 | 1 | 1 | 0 | 0 | 0 | 0 | 1 | 0 | 0 | 0 | 1 | 0 | 0 | 1 | 0 | 0 | 0 | 0 | 0 | 1 | 1 | 0 | 1 | 1 | 0 | 0 | 1 |
| 36 | Dagnra^36^ | 2014 | 1 | 1 | 1 | 0 | 1 | 1 | 0 | 0 | 0 | 1 | 0 | 0 | 0 | 1 | 1 | 0 | 0 | 0 | 0 | 1 | 0 | 0 | 1 | 1 | 0 | 1 | 1 | 0 | 0 | 0 |
| 37 | Bristow^37^ | 2015 | 1 | 0 | 1 | 1 | 1 | 1 | 0 | 0 | 0 | 1 | 0 | 0 | 0 | 1 | 1 | 0 | 0 | 0 | 0 | 0 | 0 | 0 | 1 | 1 | 0 | 1 | 1 | 0 | 0 | 1 |
| 38 | Yin^38^ | 2015 | 1 | 1 | 1 | 1 | 1 | 1 | 1 | 0 | 1 | 1 | 0 | 0 | 0 | 1 | 1 | 0 | 0 | 1 | 0 | 0 | 0 | 0 | 1 | 1 | 0 | 1 | 1 | 0 | 0 | 1 |
| 39 | Shimelis^39^ | 2015 | 1 | 1 | 1 | 1 | 1 | 1 | 1 | 1 | 0 | 1 | 0 | 0 | 1 | 1 | 0 | 0 | 0 | 0 | 1 | 1 | 0 | 0 | 1 | 1 | 0 | 1 | 1 | 0 | 0 | 1 |
| 40 | Leon^40^ | 2016 | 1 | 0 | 1 | 0 | 0 | 0 | 0 | 1 | 0 | 1 | 0 | 0 | 0 | 1 | 1 | 0 | 0 | 0 | 0 | 0 | 0 | 0 | 1 | 1 | 0 | 1 | 1 | 0 | 0 | 1 |
| 41 | Bristow^41^ | 2016 | 1 | 1 | 1 | 1 | 0 | 1 | 1 | 1 | 1 | 1 | 0 | 0 | 0 | 1 | 1 | 0 | 0 | 0 | 0 | 0 | 0 | 1 | 1 | 1 | 0 | 1 | 1 | 0 | 0 | 1 |
| 42 | Bristow^42^ | 2016 | 1 | 1 | 1 | 1 | 1 | 1 | 1 | 1 | 1 | 1 | 0 | 0 | 0 | 1 | 1 | 0 | 0 | 0 | 0 | 1 | 0 | 1 | 1 | 1 | 0 | 1 | 1 | 0 | 0 | 1 |
| 43 | Bristow^43^ | 2016 | 1 | 1 | 1 | 1 | 1 | 1 | 1 | 0 | 1 | 1 | 0 | 0 | 0 | 1 | 1 | 0 | 0 | 0 | 0 | 0 | 0 | 1 | 1 | 1 | 0 | 1 | 1 | 0 | 0 | 1 |
| 44 | Shakya^44^ | 2016 | 1 | 1 | 1 | 1 | 1 | 1 | 1 | 1 | 1 | 1 | 0 | 0 | 0 | 1 | 0 | 0 | 0 | 0 | 1 | 0 | 0 | 1 | 1 | 1 | 0 | 0 | 1 | 0 | 0 | 1 |
| 45 | Black^45^ | 2016 | 1 | 1 | 1 | 1 | 1 | 0 | 1 | 0 | 0 | 1 | 0 | 0 | 1 | 1 | 0 | 0 | 0 | 1 | 0 | 0 | 0 | 0 | 0 | 1 | 0 | 0 | 1 | 0 | 0 | 1 |
| 46 | Bowen^46^ | 2016 | 1 | 1 | 1 | 1 | 0 | 1 | 1 | 1 | 0 | 1 | 0 | 0 | 0 | 0 | 0 | 0 | 0 | 0 | 0 | 0 | 0 | 0 | 0 | 0 | 0 | 0 | 1 | 0 | 0 | 0 |
| 47 | Kalou^47^ | 2016 | 1 | 1 | 1 | 1 | 1 | 0 | 0 | 0 | 0 | 1 | 0 | 0 | 0 | 1 | 0 | 0 | 0 | 0 | 0 | 0 | 0 | 0 | 1 | 1 | 0 | 1 | 1 | 0 | 0 | 1 |

For a list of corresponding items, refer to Bossuyt *et al*.^48^

Table S2. Results of QUADAS-2 evaluation for diagnostic test accuracy evaluation studies included in the meta-analysis.

| **Study ID** | **Author** | **Year** | **Risk of bias** | | | | **Applicability concerns** | | |
| --- | --- | --- | --- | --- | --- | --- | --- | --- | --- |
|  |  |  | **Patient selection** | **Index test** | **Reference standard** | **Flow and timing** | **Patient selection** | **Index test** | **Reference standard** |
| 30 | Ondondo^30^ | 2013 | ? | ☺ | ☺ | ☺ | ☺ | ☺ | ☺ |
| 31 | Chiappe^31^ | 2013 | ? | ? | ? | ? | ☺ | ☺ | ☺ |
| 32 | Hess^32^ | 2014 | ☺ | ☺ | ? | ☺ | ☺ | ☺ | ☺ |
| 33 | Humphries^33^ | 2014 | ? | ☺ | ☺ | ☺ | ☺ | ☺ | ☺ |
| 34 | Omoding^34^ | 2014 | ☺ | ? | ? | ☺ | ☺ | ☺ | ☺ |
| 35 | Bristow^35^ | 2014 | ? | ? | ? | ? | ? | ☺ | ☺ |
| 36 | Dagnra^36^ | 2014 | ? | ? | ? | ? | ☺ | ☺ | ☺ |
| 37 | Bristow^37^ | 2015 | ? | ? | ? | ? | ☺ | ☺ | ☺ |
| 38 | Yin^38^ | 2015 | ☺ | ☺ | ? | ☺ | ☺ | ☺ | ☺ |
| 39 | Shimelis^39^ | 2015 | ☺ | ? | ? | ☺ | ☺ | ☺ | ☺ |
| 40 | Leon^40^ | 2016 | ? | ? | ? | ? | ☺ | ☺ | ☺ |
| 41 | Bristow^41^ | 2016 | ☺ | ☺ | ? | ☺ | ☺ | ☺ | ☺ |
| 42 | Bristow^42^ | 2016 | ? | ☺ | ? | ? | ☺ | ☺ | ☺ |
| 43 | Bristow^43^ | 2016 | ☺ | ☺ | ? | ☺ | ☺ | ☺ | ☺ |
| 44 | Shakya^44^ | 2016 | ☺ | ? | ? | ☺ | ☺ | ☺ | ☺ |
| 45 | Black^45^ | 2016 | ☺ | ☺ | ☺ | ☺ | ☺ | ☺ | ☺ |
| 46 | Bowen^46^ | 2016 | ☺ | ☺ | ? | ? | ☺ | ☺ | ☺ |
| 47 | Kalou^47^ | 2016 | ? | ? | ? | ? | ☺ | ☺ | ☺ |

Where ☺ = low risk, ? = unclear risk, and ☹ = high risk of bias, as stated in Whitting *et al*.^49^

**References**

30. Ondondo RO, Odoyo JB, Bukusi EA. Performance Characteristics of SD Bio Line Rapid HIV-Syphilis Duo Test Kit For Simultaneous Detection of HIV and Syphilis Infections. *Sex Transm Infect* 2013;89 (Suppl 1) A56.

31. Chiappe MA, Lopez-Torres L, Carcamo C, et al. Evaluation of a Double Rapid Test For Syphilis and HIV: SD Bioline HIV/Syphilis Duo. *Sex Transm Infect* 2013;89 (Suppl 1):A363.

32. Hess KL, Fisher DG, Reynolds GL. Sensitivity and specificity of point-of-care rapid combination syphilis-HIV-HCV tests. *PLoS One* 2014;9(11):e112190. doi: 10.1371/journal.pone.0112190

33. Humphries RM, Woo JS, Chung JH, et al. Laboratory evaluation of three rapid diagnostic tests for dual detection of HIV and Treponema pallidum antibodies. *J Clin Microbiol* 2014;52(12):4394-7. doi: 10.1128/JCM.02468-14

34. Omoding D, Katawera V, Siedner M, et al. Evaluation of the SD BIOLINE HIV/syphilis Duo assay at a rural health center in Southwestern Uganda. *BMC Res Notes* 2014;7:746.

35. Bristow CC, Adu-Sarkodie Y, Ondondo RO, et al. Multisite Laboratory Evaluation of a Dual Human Immunodeficiency Virus (HIV)/Syphilis Point-of-Care Rapid Test for Simultaneous Detection of HIV and Syphilis Infection. *Open Forum Infect Dis* 2014;1(1):ofu015. doi: 10.1093/ofid/ofu015

36. Dagnra AY, Dossim S, Salou M, et al. Evaluation of 9 rapid diagnostic tests for screening HIV infection, in Lome, Togo. *Med Mal Infect* 2014;44(11-12):525-9. doi: 10.1016/j.medmal.2014.10.007

37. Bristow CC, Leon SR, Ramos LB, et al. Laboratory evaluation of a dual rapid immunodiagnostic test for HIV and syphilis infection. *J Clin Microbiol* 2015;53(1):311-3. doi: 10.1128/JCM.02763-14

38. Yin YP, Ngige E, Anyaike C, et al. Laboratory evaluation of three dual rapid diagnostic tests for HIV and syphilis in China and Nigeria. *Int J Gynaecol Obstet* 2015;130 Suppl 1:S22-6. doi: 10.1016/j.ijgo.2015.04.004

39. Shimelis T, Tadesse E. The diagnostic performance evaluation of the SD BIOLINE HIV/syphilis Duo rapid test in southern Ethiopia: a cross-sectional study. *BMJ Open* 2015;5(4):e007371. doi: 10.1136/bmjopen-2014-007371

40. Leon SR, Ramos LB, Vargas SK, et al. Laboratory Evaluation of a Dual-Path Platform Assay for Rapid Point-of-Care HIV and Syphilis Testing. *J Clin Microbiol* 2016;54(2):492-4. doi: 10.1128/JCM.03152-15

41. Bristow CC, Leon SR, Huang E, et al. Field evaluation of a dual rapid diagnostic test for HIV infection and syphilis in Lima, Peru. *Sex Transm Infect* 2016;92(3):182-5. doi: 10.1136/sextrans-2015-052326

42. Bristow CC, Severe L, Pape JW, et al. Dual rapid lateral flow immunoassay fingerstick wholeblood testing for syphilis and HIV infections is acceptable and accurate, Port-au-Prince, Haiti. *BMC Infect Dis* 2016;16:302. doi: 10.1186/s12879-016-1574-3

43. Bristow CC, Leon SR, Huang E, et al. Field Evaluation of a Dual Rapid Immunodiagnostic Test for HIV and Syphilis Infection in Peru. *Sex Transm Dis* 2016;43(1):57-60. doi: 10.1097/OLQ.0000000000000387

44. Shakya G, Singh DR, Ojha HC, et al. Evaluation of SD Bioline HIV/syphilis Duo rapid test kits in Nepal. *BMC Infect Dis* 2016;16(1):450. doi: 10.1186/s12879-016-1694-9

45. Black V, Williams BG, Maseko V, et al. Field evaluation of Standard Diagnostics' Bioline HIV/Syphilis Duo test among female sex workers in Johannesburg, South Africa. *Sex Transm Infect* 2016 doi: 10.1136/sextrans-2015-052474

46. Bowen V, Lupoli K, Chipungu G, et al. A bundle of health- syphilis test performance in the field evaluation of a novel dual HIV/syphilis rapid test - Malawi, 2014-2015. *Sex Transm Infect* 2015;43:S223.

47. Kalou M, Castro A, Watson A, et al. Laboratory evaluation of the Chembio Dual Path Platform HIV-Syphilis Assay. *African Journal of Laboratory Medicine* 2016;5(1):A433.

48. Bossuyt PM, Reitsma JB, Bruns DE, et al. STARD 2015: an updated list of essential items for reporting diagnostic accuracy studies. *BMJ* 2015;351:h5527. doi: 10.1136/bmj.h5527

49. Whiting PF, Rutjes AW, Westwood ME, et al. QUADAS-2: a revised tool for the quality assessment of diagnostic accuracy studies. *Ann Intern Med* 2011;155(8):529-36. doi: 10.7326/0003-4819-155-8-201110180-00009
